# Maine Veterinary Medical Association

**Published:** 1897-03

**Authors:** W. L. West

**Affiliations:** Secretary


Secretary,

### MAINE VETERINARY MEDICAL ASSOCIATION.

AT the Bangor meeting we received from Prof. Charles D. Woods, Director of the Experiment Station at the Maine State College, Orono, a cordial invitation to be present the next morning and assist at a post-mortem of an interesting case.

Condition as we saw her previous to death: Dunkard Girl, an Ayrshire cow, five years old; well nourished; pulse, temperature, and respiration normal; no perceptible signs of disease.

*History.* August 14, 1895, tested highest temperature, 107.4°; August 29, 1895, 107.4°; September 4, 1895, 102.5°; September 14, 1895, 101.4°; September 26, 1895, 103°; October 8, 1895, 105°; October 18, 1895, not taken; October 31, 1895, 102.4°; November 20, 1895, 104.8°; December 7, 1895, 101.5°; January 3, 1896, 103.5°; January 10, 1896, 101.2°; January 24, 1896, 101.2°; February 20, 1896, 100.9°; July 2, 1896, 103°; August 18, 1896, 101.6°; September 16, 1896, not taken; November 3, 1896, 101.6°; January 13, 1897, 102.6°.

*Post-mortem.* The posterior mediastinal glands and the inferior external portion of the right caudal lobe were badly diseased. All other organs were healthy as far as gross appearance went.

W. L. WEST,
Secretary.

